# Post-Activation Performance Enhancement: Save Time With Active Intra-Complex Recovery Intervals

**DOI:** 10.3389/fphys.2022.840722

**Published:** 2022-07-06

**Authors:** Robert Trybulski, Piotr Makar, Dan Iulian Alexe, Silvius Stanciu, Rafał Piwowar, Michal Wilk, Michal Krzysztofik

**Affiliations:** ^1^ Provita Zory Medical Center, Zory, Poland; ^2^ Department of Medical Sciences, The Wojciech Korfanty School of Economics, Katowice, Poland; ^3^ Faculty of Physical Education, Gdańsk University of Physical Education and Sport, Gdańsk, Poland; ^4^ Faculty of Movement, Sports and Health Sciences, Vasile Alecsandri University of Bacău, Bacău, Romania; ^5^ Faculty of Food Science and Engineering, Dunarea de Jos University of Galati, Galati, Romania; ^6^ Department of Sports Training, The Jerzy Kukuczka Academy of Physical Education in Katowice, Katowice, Poland; ^7^ Institute of Sport Sciences, The Jerzy Kukuczka Academy of Physical Education in Katowice, Katowice, Poland

**Keywords:** post-activation potentiation (PAP), resistance training, explosive strength, bench press throw, upper body power

## Abstract

This study aimed to determine whether the intra-complex active recovery within the strength-power potentiating complex will impact the upper-body post-activation performance enhancement effect and how the magnitude of this effect will change across the upper-body complex training session. Thirteen resistance-trained males [the age, body mass, height, experience in resistance training, and one-repetition maximum (1RM) in bench press were 27 ± 4 years; 92.3 ± 15.4 kg; 182 ± 6 cm; 6.4 ± 2.4 years, and 118 ± 29 kg, respectively) participated in this study. Each participant completed a baseline bench press throw performance assessment at 30% 1RM. Next, five strength-power potentiating complexes consisting of a bench press at 80% 1RM were tested until the average barbell velocity decreased by 10% as a conditioning activity, and 6 min later, a re-test of bench press throw was carried out. During one experimental session during the rest interval inside the complex, they performed swiss ball leg curls, while between the complexes, a plank exercise (PAP-A) was performed. During the second experimental session, participants performed no exercises within the strength-power potentiating complexes and between them (PAP). Under control conditions, participants ran the same protocol (as the PAP condition) without the conditioning activity (CTRL). Friedman’s test showed significant differences in peak (test = 90.634; *p* < 0.0001; Kendall’s W = 0.410) and average (test = 74.172; *p* < 0.0001; Kendall’s W = 0.336) barbell velocities during bench press throw. Pairwise comparisons indicated that the peak and average barbell velocities significantly increased in the fourth set [*p* = 0.022, effect size (ES) = 0.76 and *p* = 0.013, ES = 0.69, respectively], and the average barbell velocity was also increased in the second set (*p* = 0.018, ES = 0.77) in comparison to the baseline value during the PAP-A condition. Moreover, the peak barbell velocity was increased in the second (*p* = 0.008, ES = 0.72) and third (*p* = 0.019, ES = 0.76) sets compared to the baseline value during the PAP condition. This study showed that body-weight lower-body exercise as an intra-complex active recovery did not impair the upper-body post-activation performance enhancement effect across the complex training session.

## 1 Introduction

Post-activation performance enhancement (PAPE) is a muscular phenomenon that leads to an acute improvement in power and strength performance due to the prior voluntary contractile history (Hodgson et al., 2005). This phenomenon arouses particular attention in sports that require quick and explosive actions, such as jumps, throws, or sprints (Seitz and Haff, 2016). Therefore, inducing the PAPE effect is often the purpose of a warm-up, and it is also the rationale behind using a complex training method (Freitas et al., 2017). In applied settings, this effect is caused by strength-power potentiating complexes, that is, performing a conditioning activity (CA) (usually high-loaded resistance exercise) immediately before explosive activity with a similar movement structure (i.e., back squat before countermovement jump) (Gołaś et al., 2016; Krzysztofik et al., 2020a).

One of the main factors determining the occurrence of the PAPE effect is the optimal ratio between induced fatigue and potentiation (which coexist after CA), which is essential to achieve performance enhancement (Seitz and Haff, 2016). Therefore, an adequate rest interval between the CA and the subsequent activity is critical. The CA type, volume, and intensity, as well as athlete characteristics such as experience and strength level, are cited as key factors when determining an adequate rest-interval time (Wilson et al., 2013; Seitz and Haff, 2016). Studies indicate that rest intervals lasting 2–12 min after completing the CA elicit the PAPE effect (Tsoukos et al., 2019; Tsoukos et al., 2020). Moreover, the highest efficiency is obtained within 4 and 6 min after high-intensity resistance exercise as a CA (Seitz and Haff, 2016; Krzysztofik et al., 2021b). While such a long rest interval does not appear to be a problem when inducing PAPE is the goal of a competitive warm-up, it may be impractical during an athlete’s training session—for instance, by reducing the training density and creating an opportunity to lose concentration on training (Krzysztofik et al., 2019). Furthermore, modern athletes have a limited time due to sports and nonsports duties; thus, the training process has to be highly effective. Bearing in mind the above and the undeniable benefits of resistance training, methods that show a high efficiency in increasing muscle strength and power with a high training density are particularly desirable.

Two recently published papers have proposed solutions that may increase the practicality of strength-power potentiating complex training (Lim and Barley, 2016; Lepkowski et al., 2021). Lim and Barley (2016) suggested using mobility and/or stability drills for the muscles unaffected by strength-power potentiating complex exercises as an intra-complex active recovery. A similar solution was proposed by Lepkowski et al. (2021) in the form of “delayed performance triplexes.” However, they went further and suggested inserting two exercises during intra-complex rest; one nonsimilar accessory movement like biceps curls or bent-over rows performed in between back squat and jumps and an additional less demanding one, like planks. Nevertheless, it has to be noted that Lim and Barley (2016) and Lepkowski et al. (2021) have only confined themselves to theoretical speculation and did not assess whether the introduction of these exercises would disturb the PAPE effect. Therefore, further studies need to determine if the PAPE effect would be affected when intra-complex active recovery is implemented.

Another aspect related to the low practical application to the training process from the studies on the PAPE effect is that the procedures mainly assume performing only a single set of a selected strength-power potentiating complex (Tsoukos et al., 2019; Krzysztofik et al., 2020b; Krzysztofik et al., 2020c; Krzysztofik et al., 2021a; Matusiński et al., 2021). As mentioned before, when PAPE is the goal of a warm-up, this is not a problem, but it is known that multiple sets are superior to single-set training to develop muscle strength and power (Kraemer and Ratamess, 2004). However, limited findings exist on whether the PAPE effect reported in the first set is maintained throughout the training session and to which set is sustained (Baker 2008, 2009; McLaren et al., 2017; Bauer et al., 2019). For example, McLaren et al. (2017) investigated the PAPE effect across three sets of the strength-power potentiating complex. Each consisted of four repetitions of back squats at 90% of one-repetition maximum (1RM) and four repeated 40 m sprints in college-aged males. The authors noted an improvement from one to three repetitions of the 40 m sprint in each complex set. It should also be emphasized that each complex set lasted a total of 20 min. There was a rest for as long as 16 min (8 min between exercises and 8 min between complexes), resulting in a low time efficiency of training. In another study, Bauer et al. (2019) assessed the effect of medium (six repetitions at 60% 1RM) and high-loaded (four repetitions at 90% 1RM) back squats on countermovement jump performance across three strength-power potentiating complex sets. The authors showed that PAPE was successfully induced in each of the three sets after medium and high-intensity squats. However, in the second and third sets, the level of improvement was lower. Only the study by Baker (2009) concerned the upper limbs, and the results were in agreement with McLaren et al. (2017) and Bauer et al. (2019), showing improvement in each of the three sets of bench press throw preceded by bench press with chains as a CA. However, none of these studies used more than three sets and an intra-complex active recovery.

Therefore, this study aimed to determine whether the intra-complex active recovery within the strength-power potentiating complex will impact the upper-body PAPE effect and how the magnitude of this effect will change across the upper-body complex training session. We hypothesized that implementing an intra-complex active recovery would not disturb the PAPE effect. Additionally, we thought that the PAPE effect would gradually weaken with each set.

## 2 Materials and Methods

### 2.1 Experimental Approach to the Problem

**FIGURE 1:**
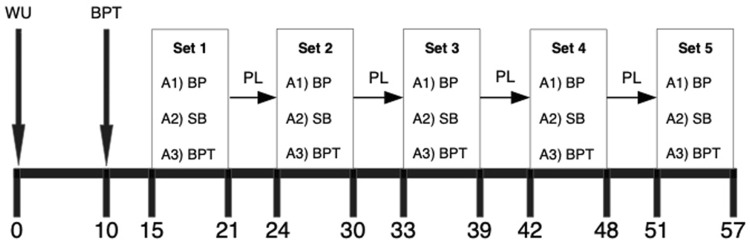
Schematic representation of the experimental session including strength-power potentiating complexes with accessory exercises. WU, warm-up; BPT, bench press throw; BP, bench press; SB, swiss ball leg curls; PL, plank.

Participants took part in a familiarization session and three experimental sessions at intervals of at least 72 h but no longer than a week. The familiarization session included the determination of the 1RM bench press, followed by two sets of the bench press at 80% 1RM performed up to a 10% loss of average barbell velocity. Then, participants made two sets of bench press throws at 30% 1RM and performed swiss ball leg curls and a plank alternately during rest intervals. Experimental sessions were conducted in random order. Each participant completed a baseline bench press throw performance assessment. Next, five strength-power potentiating complexes consisting of a bench press at 80% 1RM were tested until the average barbell velocity decreased by 10% as CA, and 6 min later, two repetitions of bench press throw at 30% 1RM were carried out. During post-activation and accessory exercise conditions (PAP-A), the participants were performing swiss ball leg curls inside the complexes and a plank exercise between them. In the post-activation potentiation condition (PAP), participants were not performing any exercises within and between the strength-power potentiating complexes. Under control conditions, participants ran the same protocol (as the PAP condition) without the CA (CTRL). Instead of this, participants rested while sitting (Figure 1). To assess the PAPE effect, the peak and average barbell velocities during bench press throws were investigated at baseline and throughout every set of workouts.

### 2.2 Subjects

Thirteen resistance-trained strength and conditioning specialist students and personal trainers were considered. Their mean ± SD, age, body mass, height, experience in resistance training, and 1RM in bench press were 27 ± 4 years, 92.3 ± 15.4 kg, 182 ± 6 cm, 6.4 ± 2.4 years, and 118 ± 29 kg, respectively. The inclusion criteria were as follows: 1) free from neuromuscular and musculoskeletal disorders, 2) at least 2 years of experience in resistance training, and 3) a self-described satisfactory health status. Participants were excluded if they reported more than 2 weeks of resistance-training absences in the past year. Moreover, participants were instructed not to perform any additional resistance exercises before testing to avoid fatigue, maintain their usual dietary and sleep habits, and not use any stimulants and alcoholic drinks throughout the study. Participants were allowed to withdraw from the experiment at any moment and informed about the benefits and potential risks of the study before providing their written informed consent for participation. Participants were not told of the expected study outcomes. The study protocol was approved by the Bioethics Committee for Scientific Research at the Academy of Physical Education in Katowice, Poland (10/2018), and performed according to the ethical standards of the Declaration of Helsinki 2013.

### 2.3 Procedures

#### 2.3.1 Familiarization Session and 1RM Strength Tests

Before the main experimental sessions, the 1RM bench press tests were performed according to the recommendations proposed by Wilk et al. (2020a) and Wilk et al. (2020b). The participants arrived in the laboratory at the same time of day as the upcoming experimental sessions (in the morning between 10:00 and 12:00 a.m.). Participants performed a standardized warm-up as described elsewhere (Krzysztofik and Wilk, 2020). Next, the participants performed 10, 8, and 4 repetitions at 30%, 50%, and 70% of their estimate, respectively. The first testing load was set to an estimated 80% 1RM and was increased by 2.5–5 kg for each subsequent attempt until the participant would not perform a lift with the proper technique. Participants were instructed to perform each repetition with a 2 s duration of the eccentric phase and maximal velocity in the concentric phase of the movement. The 1RM was defined as the highest load completed without any help from the spotters. Five-minute rest intervals were allowed between the 1RM attempts, and all 1RM values were obtained within five attempts.

Following the 1RM test, all participants performed two sets of bench press until a 10% average velocity loss at 80% 1RM, followed by two sets of two bench press throws at 30% 1RM. During rest intervals (lasting 3–4 min), participants performed swiss ball leg curls or a plank alternately.

#### 2.3.2 Experimental Sessions

In a randomized order (determined using the website randomization.com), after identical warm-up as before the 1RM tests, the participants performed three different testing conditions (at least 72 h apart, but no longer than a week) (Figure 1). The PAP condition included five strength-power potentiating complexes consisting of a bench press at 80% 1RM until the average barbell velocity decreased by 10% as CA and 6 min later two repetitions of bench press at 30% 1RM. The PAP-A condition was similar to the PAP, but between the third and fourth minutes of rest interval inside the complex, participants performed 15 repetitions of bodyweight swiss ball leg curls. However, during a 3 min rest interval between the complexes, participants performed 30 s of plank exercise (as the second min starts). In the CTRL condition, participants performed baseline and five sets of two bench press throws at 30% 1RM with 6 min rest interval. Instead of CA and accessory exercises, they rested while sitting. Hand placement on the barbell was set at 150% biacromial distance and was carefully replicated at each attempt. To assess the post-activation performance enhancement effect, peak and average barbell velocities during bench press throws were investigated at baseline and throughout every set of workouts.

#### 2.3.3 Measurement of Barbell Velocity During the Conditioning Activity

The barbell velocity during the CA was controlled using a GymAware Powertool (Kinetic Performance Technology, Canberra, Australia), a linear position transducer. The device was placed on the floor directly under the barbell, and the external end of the cable was attached to the side of the barbell. During each CA, the participants were asked to perform each repetition with a constant duration of 2 s in the eccentric phase and as fast as possible in the concentric phase. Participants were performing the bench press at 80% 1RM until the average barbell velocity decreased by 10% from the fastest repetition. This CA has been previously indicated to be effective in inducing the PAPE effect for bench press throw (Tsoukos et al., 2019, 2020). The velocity of the barbell was recorded at 50 Hz. Furthermore, the number of performed repetitions in each CA until the velocity drop was also assessed. GymAware Powertool provides reliable and valid data (Banyard et al., 2017).

#### 2.3.4 Measurement of Barbell Velocity During Bench Press Throw Exercise

In total, participants performed six sets of bench press throws at 30% 1RM on a Smith machine during each trial at baseline (5 min before the first complex) and 6 min after each CA. Before each trial, participants were instructed to execute each repetition with maximal effort, without bouncing the barbell off the chest and without intentionally pausing at the transition between the phases. The same hand placement as during bench press was replicated during each attempt. The barbell velocity during each bench press throw was evaluated using a linear position transducer Tendo Power Analyzer (Tendo Sports Machines, Trencin, Slovakia). This device is a reliable system for measuring movement velocity and power output (García-Ramos et al., 2018).

## 3 Statistical Analyses

All statistical analyses were performed using SPSS (version 25.0; SPSS, Inc., Chicago, IL, United States) and were shown as means with standard deviations (±SD). Statistical significance was set at *p* < 0.05. The normality of data distribution was checked using Shapiro–Wilk tests. However, when the normality was not confirmed, related samples’ Friedman’s two-way ANOVA by ranks was used; the effect size was estimated by Kendall’s coefficient of concordance. When significant, pairwise comparisons were also conducted using a Bonferroni test. The magnitude of mean differences was expressed with standardized effect sizes along with their 95% confidence intervals (CI); thresholds for qualitative descriptors of Hedges g were interpreted as ≤0.20 for “small,” 0.21–0.8 for “medium,” and >0.80 for “large.”

## 4 Results

### 4.1 Conditioning Activity Performance

The Shapiro–Wilk tests indicated that the normality of the data was violated for all studied variables. Friedman’s test showed significant differences in peak (test = 28.548; *p* = 0.001; Kendall’s W = 0.244) and average (test = 76.443; *p* < 0.0001; Kendall’s W = 0.653) barbell velocities and the number of performed repetitions (test = 53.465; *p* < 0.0001; Kendall’s W = 0.457) (Table 1).

**TABLE 1:** Comparison of conditioning activity (bench press) performance between conditions.

	Set 1 (95 CI)	Set 2 (95 CI)	Set 3 (95 CI)	Set 4 (95 CI)	Set 5 (95 CI)
Peak Barbell Velocity (m/s)					
PAP-A	0.65 ± 0.05 (0.62–0.68)	0.72 ± 0.07* (0.67–0.76)	0.69 ± 0.06 (0.65–0.72)	0.67 ± 0.07 (0.63–0.72)	0.63 ± 0.08 (0.59–0.68)
PAP	0.66 ± 0.04 (0.64–0.69)	0.72 ± 0.05* (0.68–0.75)	0.69 ± 0.06 (0.65–0.73)	0.68 ± 0.09 (0.63–0.74)	0.66 ± 0.08 (0.61–0.71)
Average Barbell Velocity (m/s)					
PAP-A	0.46 ± 0.03 (0.44–0.48)	0.52 ± 0.04* (0.49–0.55)	0.49 ± 0.05* (0.46–053)	0.43 ± 0.04^#^ (0.41–0.45)	0.38 ± 0.04 (0.35–0.41)
PAP	0.47 ± 0.03 (0.45–0.49)	0.54 ± 0.06* (0.5–0.57)	0.5 ± 0.05* (0.47–0.54)	0.45 ± 0.04^#^ (0.42–0.47)	0.39 ± 0.05 (0.35–0.42)
Repetitions (n)					
PAP-A	4.3 ± 1.3 (3.6–5.1)	4.5 ± 1* (3.9–5)	3.9 ± 0.9 (3.4–4.4)	3.4 ± 0.9 (2.9–3.9)	2.9 ± 0.7 (2.4–3.3)
PAP	4.4 ± 0.8* (3.9–4.8)	4.2 ± 0.9* (3.7–4.8)	3.7 ± 1.1 (3–4.4)	3.0 ± 0.6^†^ (2.7–3.3)	2.7 ± 0.6 (2.3–3.1)

Results are mean ± SD (95% confidence intervals); *significantly different in comparison to Set 5; ^#^significantly different in comparison to Set 2; ^†^significantly different in comparison to Set 1; PAP-A, post-activation and accessory exercise condition; PAP, post-activation condition.

Pairwise comparisons indicated that the peak barbell velocity significantly decreased in the fifth set compared to the second (*p* = 0.009; ES = 1.16) during the PAP-A condition. Moreover, the peak barbell velocity was significantly lower in the fifth set during the PAP-A condition than in the 2nd set (*p* = 0.009; ES = 0.87) during the PAP condition.

Pairwise comparisons demonstrated that the average barbell velocity significantly decreased in the fifth set in comparison to the second (*p* < 0.001; ES = 3.39) and third (*p* < 0.001; ES = 2.35) sets and in the fourth set compared to the second (*p* = 0.007; ES = 2.18) during the PAP-A condition. Similarly, there were significant decreases in average barbell velocity in the fifth set in comparison to the second (*p* < 0.001; ES = 2.63) and third (*p* = 0.001; ES = 2.13) sets and in the fourth when compared to the second (*p* = 0.048; ES = 1.28) during the PAP condition.

Pairwise comparisons indicated that the number of performed repetitions significantly decreased in the fifth set in comparison to the second (*p* = 0.015; ES = 1.8) during the PAP-A condition. Moreover, the number of performed repetitions significantly decreased in the fifth set in comparison to the first (*p* = 0.001; ES = 2.33) and second (*p* = 0.009; ES = 1.88) sets and in the fourth compared to the first (*p* = 0.013; ES = 1.92) during the PAP condition.

### 4.2 Bench Press Throw Performance

The Shapiro–Wilk tests indicated that the normality of the data was violated for both peak and average barbell velocities. Friedman’s test showed significant differences in peak (test = 90.634; *p* < 0.0001; Kendall’s W = 0.410) and average (test = 74.172; *p* < 0.0001; Kendall’s W = 0.336) barbell velocities (Table 2).

**TABLE 2:** Comparison of bench press throw performance between conditions.

	BA (95 CI)	Set 1 (95 CI)	Set 2 (95 CI)	Set 3 (95 CI)	Set 4 (95 CI)	Set 5 (95 CI)
Peak Barbell Velocity (m/s)						
PAP-A	1.97 ± 0.12 (1.9–2.04)	2.06 ± 0.13 (1.98–2.14)	2.05 ± 0.11 (1.98–2.12)	2.04 ± 0.12 (1.97–2.11)	2.06 ± 0.11* (1.99–2.13)	2.01 ± 0.13 (1.94–2.1)
PAP	1.98 ± 0.11 (1.92–2.05)	2.06 ± 0.11 (1.99–2.13)	2.07 ± 0.13* (1.99–2.15)	2.07 ± 0.12* (1.99–2.14)	2.04 ± 0.11 (1.98–2.11)	2.03 ± 0.12 (1.96–2.1)
CTRL	1.97 ± 0.14 (1.89–2.06)	1.99 ± 0.12 (1.92–2.07)	1.98 ± 0.13 (1.91–2.06)	1.97 ± 0.16 (1.88–2.07)	1.97 ± 0.15 (1.88–2.06)	2 ± 0.17 (1.9–2.1)
Average Barbell Velocity (m/s)						
PAP-A	1.24 ± 0.07 (1.2–1.29)	1.29 ± 0.07 (1.25–1.33)	1.3 ± 0.08* (1.25–1.35)	1.29 ± 0.07 (1.25–1.33)	1.29 ± 0.07*^#^ (1.26–1.34)	1.27 ± 0.08 (1.23–1.32)
PAP	1.25 ± 0.06 (1.22–1.29)	1.28 ± 0.05 (1.25–1.31)	1.3 ± 0.06 (1.26–1.33)	1.28 ± 0.05 (1.26–1.31)	1.3 ± 0.06 (1.25–1.33)	1.28 ± 0.04 (1.26–1.32)
CTRL	1.24 ± 0.09 (1.18–1.3)	1.25 ± 0.09 (1.2–1.3)	1.25 ± 0.08 (1.2–1.3)	1.24 ± 0.09 (1.19–1.3)	1.23 ± 0.08 (1.18–1.28)	1.25 ± 0.07 (1.21–1.3)

Results are mean ± SD (95% confidence intervals); *significant increase in comparison to baseline; ^#^significant increase in comparison to the corresponding set in the CTRL condition; BA, baseline; PAP-A, post-activation and accessory exercise condition; PAP, post-activation condition; CTRL, control condition.

Pairwise comparisons indicated that the peak barbell velocity significantly increased in the fourth set (*p* = 0.022; ES = 0.76) compared to the baseline value during the PAP-A condition. Moreover, a significant increase in peak barbell velocity were noted in the second (*p* = 0.008; ES = 0.72) and third (*p* = 0.019; ES = 0.76) sets compared to the baseline value during the PAP condition. No statistically significant differences in the corresponding sets between the conditions and baseline and the following sets during the CTRL condition were found.

Pairwise comparisons demonstrated that the average barbell velocity significantly increased in the second (*p* = 0.018; ES = 0.77) and fourth sets (*p* = 0.013; ES = 0.69) compared to the baseline value during the PAP-A condition. Furthermore, a significantly higher average barbell velocity was found in the fourth set during PAP-A than in the fourth set during the CTRL condition (*p* = 0.007; ES = 0.77). No statistically significant differences between the baseline and following sets during PAP and CTRL conditions were found.

## 5 Discussion

This study aimed to determine whether the use of intra-complex active recovery within the strength-power potentiating complexes will impact the upper-body PAPE effect and how the magnitude of this effect will change across the upper-body complex training session. The results showed that body-weight lower-body exercise as an intra-complex active recovery did not impair the upper-body PAPE effect across the complex training session. On the contrary, a significant enhancement of the peak and average barbell velocities was reported in the fourth set and the average barbell velocity in the second set compared to the baseline value.

This study indicated that applying exercises to a body region other than the one involved in the strength-power potentiating complex will not harm the PAPE effect. Alternating sets of resistance exercises involving other body regions or opposing muscle groups is a practice that has long been used in resistance training and has been identified as an effective way to reduce exercise duration without impairment in performance (Baker and Newton, 2005; Robbins et al., 2010; Weakley et al., 2017; Krzysztofik et al., 2019). For instance, Weakley et al. (2017) showed that it supersets efficiency and reduces the training time compared to traditional training sessions. Furthermore, a smaller decrease in jump height was reported immediately after a workout performed in a superset manner than conventional. However, the study by Baker and Newton (2005) reported an increase in bench press throw power output when it was performed alternately with bench pull. The current study’s findings also confirm that if the introduced exercise involves other body regions, it will not affect the PAPE effect.

The exact mechanisms underlying the PAPE effect are still unknown. However, the existing literature is consistently indicating that this phenomenon is related to local physiological responses (Hodgson et al., 2005; Tillin and Bishop, 2009; Vandenboom, 2016) and reveals that enhancement in performance depends on the balance between fatigue and the potentiation state (Seitz and Haff, 2016). The results of the current study indirectly support these statements. Despite implementing lower-body activity within an upper-body strength-power potentiating complex, bench press throw performance enhancement was still noticed. Therefore, it is plausible to speculate that active rest used within the complex that engages muscles not involved in CA and post-CA tasks does not disturb the advantage of a potentiation state over fatigue in the muscles stimulated by used CA. These findings provide valuable practical knowledge for coaches and practitioners about complex training programming. First, implementing activities within a complex set enhances the training efficiency by actively using the rest interval within the complex. These can be, for example, compensation exercises, mobility, or stability drills that address the player’s needs and still benefit from improving physical fitness *via* the PAPE effect. Thanks to this, there will be no need to perform separate training units of this type, and this time can be devoted to improving other athletic aspects. However, our study found this approach effective for upper–lower–upper body exercise, but it is uncertain whether it would also be similar in other combinations. Furthermore, research is required to establish this.

Another critical aspect of high practical importance was determining whether the PAPE effect would be maintained during multiple sets of the strength-power potentiating complex. According to the authors’ knowledge, this study was the second one investigating acute changes in power performance across multiple sets of upper body complex training sessions (Baker, 2009). Furthermore, it is the first one in which more than three sets were used in training sessions. As in earlier studies (Baker and Newton, 2005; Baker, 2009; McLAREN et al., 2017; Bauer et al., 2019), the PAPE effect was also maintained in three sets of the complex. However, the current study found sustained improvement throughout the training session, which reached statistical significance in the second and fourth sets. It partially contradicts the findings of Bauer et al. (2019), who found that the levels of improvement were lower in the second and third sets. It seems to be related to the CA volume used. Bauer et al. (2019) evaluated the effect of medium- (six reps at 60% 1RM) and high-intensity (four reps at 90% 1RM) squats on CMJ performance, and this study used individual volume selection for each athlete based on a 10% drop in average barbell velocity during CA. Hence, bearing in mind the decisive influence of the fatigue-potentiation relationship on the PAPE effect, it can be assumed that the decreasing effect in Bauer et al. (2019) was associated with the accumulation of fatigue due to the type of used CA.

In the long-term training programming process, three sets are usually planned in the first weeks, and as progression is introduced, the intensity or volume of training increases, for example, to four and then five sets at the end (Kraemer and Ratamess, 2004). The results of this study provided information that the PAPE effect was maintained up to the fifth set. Therefore, based on the results of this study, it seems that even five sets of upper body complexes can be used in a training session. However, we are not sure if similar results would have been obtained if the number of repetitions in each set had been fixed instead of velocity control. In this study, the number of repetitions in the CA was adjusted *via* velocity loss in each set and decreased with each successive set. Interestingly, an increase in the average barbell velocity was also noted during CA. Therefore, it indicates that a 9 min rest interval between consecutive CAs is sufficient to avoid fatigue accumulation, and the PAPE effect is sustained when velocity control is used.

The results of this study should be interpreted in light of certain limitations. First, only one kind of CA was evaluated (one intensity and velocity loss were applied with a single rest time interval); thus, caution is needed in exploring these findings in other protocols. Furthermore, although the participants were experienced in resistance training, according to Seitz and Haff’s classification (Seitz and Haff, 2016), they were considered weak individuals. Therefore, these results should be carefully interpreted concerning competitors with a high level of strength. Moreover, any physiological mechanisms were not assessed as the basis for the PAPE effect. Hence, we cannot be sure about what mechanisms contributed to the reported improvement in performance. Furthermore, an evaluation was made only during a single training session; it is not certain that the effects will improve performance after long-term use.

This study provides valuable practical implications for coaches and practitioners regarding programming complex training. Based on these results, we can conclude that implementing body-weighted lower body exercises and stability within the strength-power potentiating complex did not attenuate the PAPE effect. Therefore, the programming of low-intensity activities within the strength-power potentiating complex may be a solution to successfully reap the benefits of the PAPE effect without unnecessarily extending the training time. In addition, when a high-loaded bench press with velocity control as a CA is used, the PAPE effect is maintained across five consecutive strength-power potentiating complex sets. Thus, we recommended using three to five sets of upper body strength-power potentiating complexes, interspersed with low-intensity lower body or stabilizing exercises.

## Data Availability

The raw data supporting the conclusion of this article will be made available by the authors, without undue reservation.
